# Improved hepatic arterial fraction estimation using cardiac output correction of arterial input functions for liver DCE MRI

**DOI:** 10.1088/1361-6560/aa553c

**Published:** 2017-01-25

**Authors:** Manil D Chouhan, Alan Bainbridge, David Atkinson, Shonit Punwani, Rajeshwar P Mookerjee, Mark F Lythgoe, Stuart A Taylor

**Affiliations:** 1 Division of Medicine, University College London (UCL) Centre for Medical Imaging, UCL, London, UK; 2 Department of Medical Physics, University College London Hospitals NHS Trust, London, UK; 3 Division of Medicine, University College London (UCL) Institute for Liver and Digestive Health, UCL, London, UK; 4 Division of Medicine, University College London (UCL) Centre for Advanced Biomedical Imaging, UCL, London, UK; stuart.taylor1@nhs.net

**Keywords:** liver DCE MRI, arterial input functions, cardiac output, pharmacokinetic modelling

## Abstract

Liver dynamic contrast enhanced (DCE) MRI pharmacokinetic modelling could be useful in the assessment of diffuse liver disease and focal liver lesions, but is compromised by errors in arterial input function (AIF) sampling. In this study, we apply cardiac output correction to arterial input functions (AIFs) for liver DCE MRI and investigate the effect on dual-input single compartment hepatic perfusion parameter estimation and reproducibility.

Thirteen healthy volunteers (28.7  ±  1.94 years, seven males) underwent liver DCE MRI and cardiac output measurement using aortic root phase contrast MRI (PCMRI), with reproducibility (*n*  =  9) measured at 7 d. Cardiac output AIF correction was undertaken by constraining the first pass AIF enhancement curve using the indicator-dilution principle. Hepatic perfusion parameters with and without cardiac output AIF correction were compared and 7 d reproducibility assessed.

Differences between cardiac output corrected and uncorrected liver DCE MRI portal venous (PV) perfusion (*p*  =  0.066), total liver blood flow (TLBF) (*p*  =  0.101), hepatic arterial (HA) fraction (*p*  =  0.895), mean transit time (MTT) (*p*  =  0.646), distribution volume (DV) (*p*  =  0.890) were not significantly different. Seven day corrected HA fraction reproducibility was improved (mean difference 0.3%, Bland–Altman 95% limits-of-agreement (BA95%LoA)  ±27.9%, coefficient of variation (CoV) 61.4% versus 9.3%, ±35.5%, 81.7% respectively without correction). Seven day uncorrected PV perfusion was also improved (mean difference 9.3 ml min^−1^/100 g, BA95%LoA  ±506.1 ml min^−1^/100 g, CoV 64.1% versus 0.9 ml min^−1^/100 g, ±562.8 ml min^−1^/100 g, 65.1% respectively with correction) as was uncorrected TLBF (mean difference 43.8 ml min^−1^/100 g, BA95%LoA  ±586.7 ml min^−1^/ 100 g, CoV 58.3% versus 13.3 ml min^−1^/100 g, ±661.5 ml min^−1^/100 g, 60.9% respectively with correction). Reproducibility of uncorrected MTT was similar (uncorrected mean difference 2.4 s, BA95%LoA  ±26.7 s, CoV 60.8% uncorrected versus 3.7 s, ±27.8 s, 62.0% respectively with correction), as was and DV (uncorrected mean difference 14.1%, BA95%LoA  ±48.2%, CoV 24.7% versus 10.3%, ±46.0%, 23.9% respectively with correction).

Cardiac output AIF correction does not significantly affect the estimation of hepatic perfusion parameters but demonstrates improvements in normal volunteer 7 d HA fraction reproducibility, but deterioration in PV perfusion and TLBF reproducibility. Improved HA fraction reproducibility maybe important as arterialisation of liver perfusion is increased in chronic liver disease and within malignant liver lesions.

## 1. Introduction

Liver dynamic contrast enhanced (DCE) MRI has been used to investigate diffuse parenchymal changes in fibrosis/cirrhosis (Annet *et al* 2003, Hagiwara *et al* 2008, Kim *et al* 2008), but also in the characterisation of focal liver lesion vascularity and quantification of tumour angiogenesis (Jackson *et al* 2002). The technique involves acquisition of high temporal resolution images following intravenous administration of a gadolinium-based contrast agent (CA). Dynamic changes in tissue signal intensity (SI) are recorded, converted into CA concentration, with quantification of tissue perfusion using pharmacokinetic modelling (Tofts and Kermode 1991, Materne *et al* 2002, Pandharipande *et al* 2005).

Pharmacokinetic modelling requires regions-of-interest (ROIs) to be placed over dynamically imaged afferent vessels to derive vascular input function (VIFs). These are measured following a rate-controlled injection, ideally directly into the afferent vessel and as close as possible to the organ of interest. VIFs are then convolved with tissue enhancement curves to derive inflow and outflow constants that reflect perfusion.

In clinical practice, VIF sampling takes place away from both the injection site (contrast usually given via peripheral vein) and the organ of interest. VIFs are therefore widened by dilution and patient-specific circulatory factors such as cardiac output. In addition to this sampling of arterial input function (AIFs) using MRI can be particularly challenging. Sampling of rapid changes in CA concentration in high flow vessels can result in dephasing effects (Utz *et al* 2008, Oechsner *et al* 2009), high arterial flow velocities result in inflow effects (Peeters *et al* 2004), pulsatile flow results in artefactual loss of signal, small vessel/ROI sizes result in partial voluming (van der Schaaf *et al* 2006) and limitations in the temporal resolution of the acquisition can omit essential AIF features (Gill *et al* 2014). All of these factors can result in erroneous AIF sampling. Pharmacokinetic modelling using inaccurate AIFs thus introduces errors in estimated hepatic perfusion parameters.

Liver DCE MRI has the additional complexity of dual portal venous (PV) and hepatic arterial (HA) blood supply, necessitating measurement of both an AIF and portal venous input function (PVIF). Sampling of PVIFs is less troublesome than AIFs, as the PV demonstrates slower flow, slower rates of CA concentration change and lower peak CA concentration.

Previously, Zhang *et al* (2009) have proposed using MR measurements of cardiac output to correct AIFs in the measurement of renal perfusion. Using indicator-dilution theory: the principle that the volume of a compartment can be estimated from knowledge of the concentration and volume of indicator introduced into a circulatory system, Zhang *et al* used cardiac output measurements to correct the area under the AIF peak. They applied their method to demonstrate improved precision and repeatability of estimated renal perfusion parameters (Zhang *et al* 2009). It is unknown if these benefits apply to more complex organs such as the liver. We hypothesise that the use of cardiac output corrected AIFs in DCE MRI pharmacokinetic modelling would significantly influence hepatic perfusion parameter quantification, and improve their reproducibility.

The purpose of the study was therefore to apply the principal of cardiac output correction of AIFs described by Zhang *et al* to estimate hepatic DCE MRI perfusion parameters using the dual input single compartment model (Materne *et al* 2000, 2002, Miyazaki *et al* 2008) in healthy volunteers and to evaluate the effects of cardiac output corrected AIFs on DCE MRI hepatic perfusion parameters and their 7 d reproducibility.

## 2. Materials and methods

### 2.1. Subjects and preparation

The study was approved by the Local Ethics Committee and all participants provided informed written consent. Advertisement within the university campus was used to recruit volunteers who were eligible only if (a) they had no contraindications to MRI, (b) they were not taking any long-term medication (excluding oral contraception) and (c) had no past history of gastrointestinal or liver disease. Fourteen volunteers were recruited, but one was excluded due to claustrophobia. Seven males (aged 26.5  ±  1.36 years) and six females (aged 31.2  ±  2.62 years) participated in the study. All subjects fasted for 6 h before the MRI scan and avoided caffeinated fluids. A peripheral upper limb vein was cannulated (19G) in preparation for administration of intravenous contrast. Nine subjects consented to be re-scanned 7 d after the original scan for reproducibility studies. These subjects followed identical preparation and MRI protocol, and were scanned at a comparable time of day as the first study (within 2 h). This cohort has previously been described (Chouhan *et al* 2016a), where the effects of altering VIF CA bolus arrival delays on liver DCE MRI perfusion parameters were investigated. The current study presents new data on cardiac output correction of AIFs.

### 2.2. DCE MRI

Imaging was performed using a 3.0T scanner (Achieva, Philips Healthcare, Best, Netherlands) using a 16 channel body coil (SENSE XL-Torso, Philips Healthcare, Best, Netherlands) as previously described (Chouhan *et al* 2016a). Briefly, anatomical imaging using a breath hold balanced steady-state free precession (SSFP) sequence was used to plan DCE studies for inclusion of the liver, retroperitoneal vessels and heart. T1 measurements were obtained using multi-flip angle (5, 7, 10, 15 and 20°) three-dimensional (3D) gradient echo imaging, with *B*_1_ non-uniformity correction (Treier *et al* 2007). 3D gradient turbo field echo (TFE) imaging with spectral attenuated inversion recovery (SPAIR) fat suppression was used for coronal plane DCE imaging. Sixty slices were obtained from each 15 cm volume within 3.35 s, with sequential scanning for 5 min (sequence parameters given in table 1). Ten ml of Gd-DOTA (gadoterate dimeglumine, Dotarem^®^, Guerbet, Roissy, France), diluted in 10 ml of normal saline, was injected after the first five volumes were acquired at 4 ml/s (Spectris^®^, Medrad Inc., USA), followed by a 20 ml saline flush. The first breath hold instruction was given before the CA injection and subjects thereafter continued self-directed expiration breath holds for the remainder of the DCE study.

**Table 1.** Sequence parameters.

	T1 multi-flip angle	B1 map	DCE MRI	PCMRI
TR/TE (seconds)	4.0/2.0	100/1.0	2.0/1.0	8.70/5.22
Flip angle (°)	5, 7, 10, 15, 20	60	10	10
Matrix size (pixels)	240 × 240	100 × 100	240 × 240	336 × 336
Field-of-view (mm)	475 × 475	475 × 475	475 × 475	271 × 210
Spatial resolution (mm^2^)	1.98 × 1.98	4.75 × 4.75	1.98 × 1.98	0.808 × 0.625
Bandwidth (Hz/pixel)	389	1447	1411	210
Slice thickness (mm)	5	5	5	5
Slice gap (mm)	2.5	5	2.5	—
Slices per volume	60	30	60	—

### 2.3. Cardiac output measurement using phase contrast MRI

Cardiac output was measured at the aortic root. The study coordinator (Blind, radiology research fellow with 5 years’ experience in abdominal imaging) planned 2D cine PCMRI with expiratory breath-hold and retrospective cardiac gating using the previously acquired anatomical SSFP images. Planning was undertaken in two planes to ensure orthogonality to the aortic root (table 1). Studies were performed using a velocity encoding (${{V}_{\text{enc}}}$) setting of 120 cm s^−1^, and images were reviewed for aliasing with ${{V}_{\text{enc}}}$ settings increased when appropriate. PCMRI measurements were performed using seven phases through the cardiac cycle and acquired three times in succession, over three breath holds. Aortic root PCMRI studies were repeated after 7 d for reproducibility studies.

### 2.4. Post-processing

Post-processing was performed using Matlab code (MathWorks, Natick, USA) developed in house as described previously (Chouhan *et al* 2016a). Briefly, motion artefacted DCE volumes were discarded—no VIF peaks were missed. Five coronal slices each separated by 10 mm and centred around the portal vein, were selected for analysis. Slices were matched to previously derived T1 maps and robust data decomposition registration was applied to correct for tissue displacement and deformation (Hamy *et al* 2014). Where SI data was missing due to discarded volumes, these were estimated using linear interpolation. SI maps were then converted on a pixel-wise basis into CA concentration maps for each of the five slices (Aronhime *et al* 2014, Gill *et al* 2014). Hepatic parenchymal ROIs were positioned to avoid major inflow or outflow vessels, firstly in the right upper region (segments VII/VIII), left liver (segments II/III) and right lower region (segments V/VI). Three ROIs were positioned on each of the five slices. For each VIF, ROIs were also placed in the left ventricle chamber and PV as demonstrated previously (Chouhan *et al* 2016a). Perfusion parameters (detailed below) extracted from all fifteen ROIs (three ROIs on five slices) were averaged across all subjects for comparative studies.

Aortic root flow was quantified from PCMRI data using freely available software (Segment, Medviso, Lund, Sweden) and multiplied by the contemporaneous heart rate to estimate cardiac output. The mean of triplicate cardiac output measurements was used for analysis. All post-processing was undertaken by the study coordinator.

### 2.5. AIF correction using cardiac output measurements

Based on the method described by Zhang *et al* (2009), AIFs expressed as the concentration of CA as function of time (${{C}_{\text{a}}}(t)$), were converted back into their raw SI curves (${{S}_{\text{a}}}(t)$). The peak and main decline in concentration of the raw SI AIF was then extrapolated and fitted with the gamma variate function to derive an expression for first pass SI (${{S}_{\text{fp}}}(t)$) (Davenport 1983):

1 \begin{eqnarray*}{{S}_{\text{fp}}}(t)=~{{S}_{0}}+A{{\left(t-{{\tau}_{\text{a}}}\right)}^{\alpha}}{{\text{e}}^{-\left(t-{{\tau}_{a}}\right)/\beta}}\end{eqnarray*}

where, ‘${{S}_{0}}$’ represents baseline SI, ‘${{\tau}_{\text{a}}}$’ is the AIF delay and ‘$A$’, ‘$\alpha $’ and ‘$\beta $’ are fitted parameters. The first pass SI curve (${{S}_{\text{fp}}}(t)$) can then be converted back into a first pass AIF CA curve (${{C}_{\text{fp}}}(t)$). Using the indicator-dilution principle:

2 \begin{eqnarray*}{\int}^{}{{C}_{\text{fp}}}(t)~\text{d}t=\frac{D}{Q}\end{eqnarray*}

where, ‘$D$’ is the mass of injected extracellular CA and ‘$Q$’ is the bulk flow. As ‘$D$’ is known and ‘$Q$’ was measured independently using PCMRI aortic root flow, ‘${{C}_{\text{fp}}}(t)$’ area under the curve (AUC) could be adjusted to represent the expected first pass CA concentration curve based on known cardiac output. This adjusted first pass CA concentration curve could then be converted back into corrected raw first pass SI data. The converted SI curves could then be used to derive a new estimate for baseline SI ($S_{0}^{\prime}$).

In the final steps, the raw SI curves for the entire AIF (including the recirculated portion) are shifted a new baseline ($S_{0}^{\prime}$). The new corrected raw SI AIF is then converted back to a corrected AIF CA concentration curve ($C_{\text{a}}^{\prime}(t)$), ready for use in pharmacokinetic modelling. AIF correction factors were derived by expressing the area under the $C_{\text{a}}^{\prime}(t)$ curve as a percentage of the area under the ${{C}_{\text{a}}}(t)$ curve.

### 2.6. Pharmacokinetic modelling

Dual-input single compartment modelling was undertaken as reported previously (Materne *et al* 2002, Hagiwara *et al* 2008). Briefly, liver parenchymal CA concentration as a function of time (${{C}_{\text{L}}}(t)$) can be expressed as:

3 \begin{eqnarray*}{{C}_{\text{L}}}(t)={\int}_{0}^{t}\left[{{k}_{1\text{a}}}{{C}_{\text{a}}}\left({{t}^{'}}-~{{\tau}_{\text{a}}}\right)+{{k}_{1\text{p}}}{{C}_{\text{p}}}\left({{t}^{'}}-{{\tau}_{\text{p}}}\right)\right]{{\text{e}}^{-{{k}_{2}}\left(t-{{t}^{'}}\right)}}~\text{d}{{t}^{\prime}}\end{eqnarray*}

where ${{C}_{\text{a}}}(t)$ represents the arterial input CA concentration as a function of time, ${{C}_{\text{p}}}(t)$ represents the PV input CA concentration as a function of time, ${{k}_{1\text{a}}}$ represents the arterial inflow constant, ${{k}_{1\text{p}}}$ represents the PV inflow constant, ${{k}_{2}}$ represents the outflow constant, ${{\tau}_{\text{a}}}$ represents the delay between the arrival of CA in the AIF and parenchymal ROIs and ${{\tau}_{\text{p}}}$ represents the delay between arrival of CA in the PVIF and parenchymal ROIs. Model fitting was undertaken firstly using pre-estimation of CA bolus arrival delays with constrained free modelling (Chouhan *et al* 2016a), followed by non-linear least squares fitting with in house developed Matlab code. Inflow and outflow constants were used to derive estimates of PV perfusion (ml min^−1^/100 g), total liver blood flow (TLBF, sum of HA and PV perfusion, ml min^−1^/100 g), HA fraction (%), distribution volume (DV, %) and mean transit time (MTT, seconds) as reported previously (Materne *et al* 2002, Hagiwara *et al* 2008). Pharmacokinetic modelling was undertaken using both the measured (${{C}_{\text{a}}}(t)$) and corrected AIF ($C_{\text{a}}^{\prime}(t)$) and the residual sum of squares was recorded as a measure of model fitting.

### 2.7. Statistical analysis

Kolmogorov–Smirnov tests were used to confirm the normality of variable distributions. Paired *t*-tests/Wilcoxon matched pairs signed rank tests were used to compare perfusion parameters derived from cardiac output corrected and uncorrected AIF data. Seven day reproducibility studies were assessed using Bland–Altman (BA) analysis of agreement, with calculation of the mean difference (bias), 95% limits of agreement (LoA) and coefficients of variation and compared for cardiac output corrected and uncorrected AIF data. Data was expressed as mean  ±  standard error and statistical significance was assigned at *p*  <  0.05.

## 3. Results

### 3.1. Cardiac output correction of AIFs for estimation of hepatic perfusion parameters

**Figure 1.**
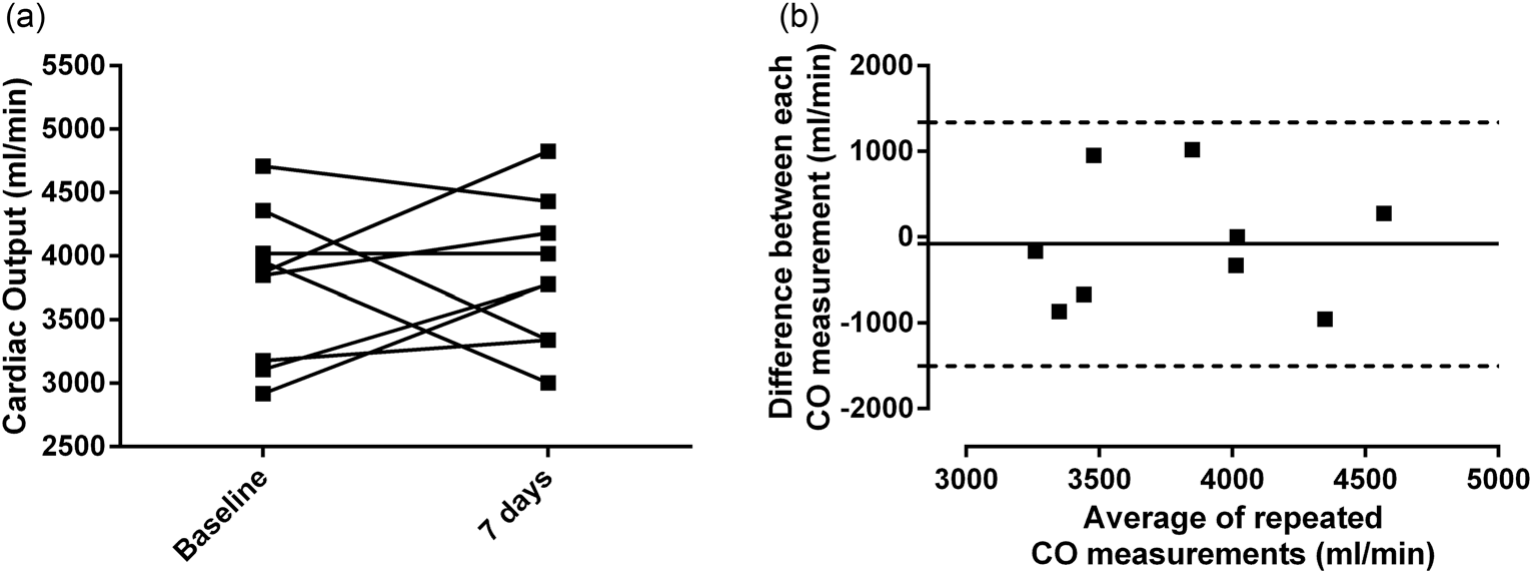
Pairwise changes in cardiac output and 7 d reproducibility. Cardiac output measured at baseline and after 7 d, (a) as a ladder chart to demonstrate pairwise changes (*p*  =  0.7450) and (b) for Bland–Altman reproducibility analysis.

**Figure 2.**
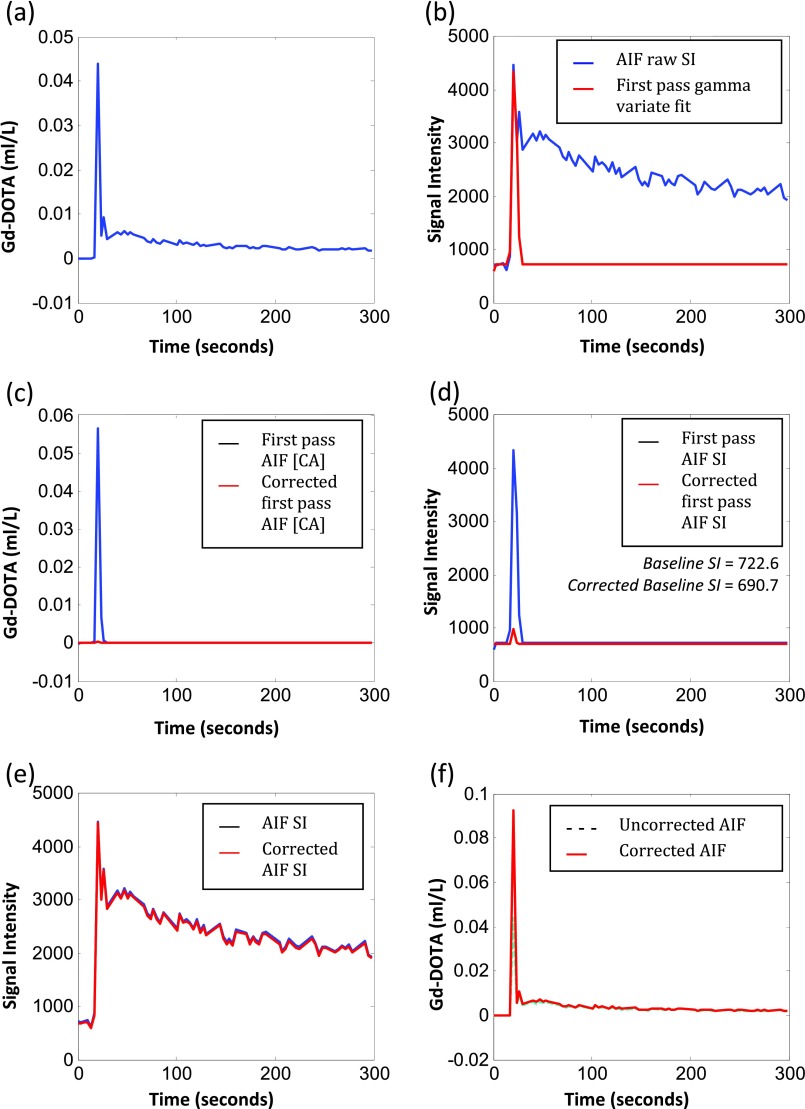
Cardiac output AIF correction. The uncorrected AIF (a), is converted back into raw signal intensity ((b), blue curve). The first past portion of the SI curve is modelled using the gamma variate function ((b), red curve). The SI gamma variate function is then converted back to derive a first pass CA concentration curve ((c), blue curve) and adjusted using cardiac output data ((c), red curve). This is then converted back into raw SI data (d). The corrected first-pass curve will provide an alternate estimate for baseline SI. The original AIF raw SI curve is then adjusted to the new baseline (e). The new adjusted curve is used to derive a corrected AIF CA concentration curve ((f), red curve).

**Figure 3.**
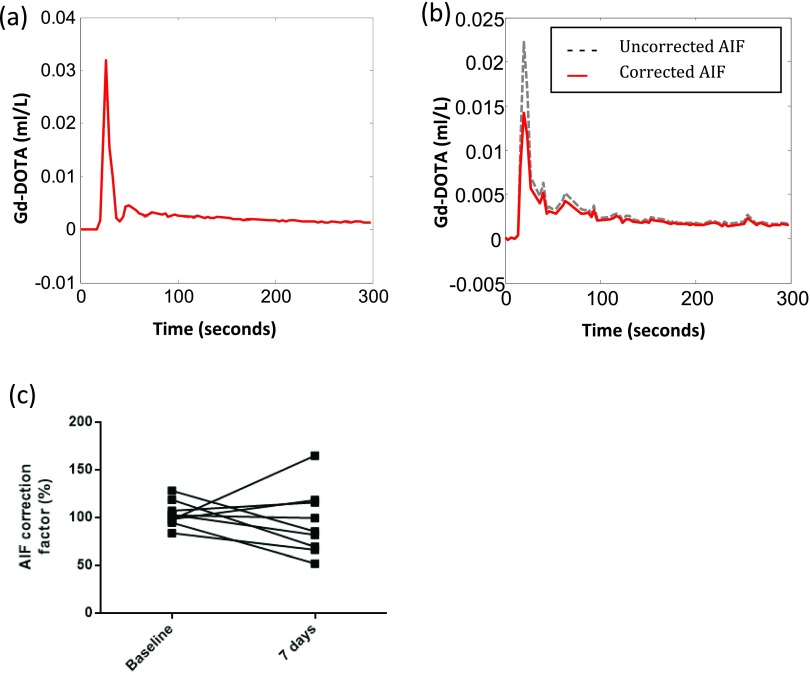
Effects of cardiac output correction on AIF size. On both diagrams, the corrected AIF is shown in red and the uncorrected AIF is show in dashed green. In some instances, correction resulted in little or no change to the AIF itself, as shown by the dataset in (a), some cases (*n*  =  11) demonstrated a reduction in AIF size (b), while others demonstrated an increase (*n*  =  11) as shown by the dataset in figure 1(f). Pairwise changes in AIF correction factor at baseline and after 7 d (*p*  =  0.4931) are shown in (c).

Across the cohort, mean cardiac output was 4143  ±  148 ml min^−1^, ranging from 2918 to 5359 ml min^−1^. The mean difference for repeated cardiac output measurements (*n*  =  9) was 82.23 ml min^−1^ (figure 1), with BA 95% LoAs of  ±1358 ml min^−1^ and coefficients of variation of 18.48% (inter-subject) and 5.85% (intra-subject). Figure 2 illustrates the uncorrected AIF, uncorrected and first pass AIF SI curve with gamma variate fit, corrected first pass CA concentration curve and corrected AIF from a sample data set. Mean AIF correction factor was 98.12  ±  5.34% (range 52.05–165.1%, coefficient of variation (CoV) 25.54% figure 3).

**Figure 4.**
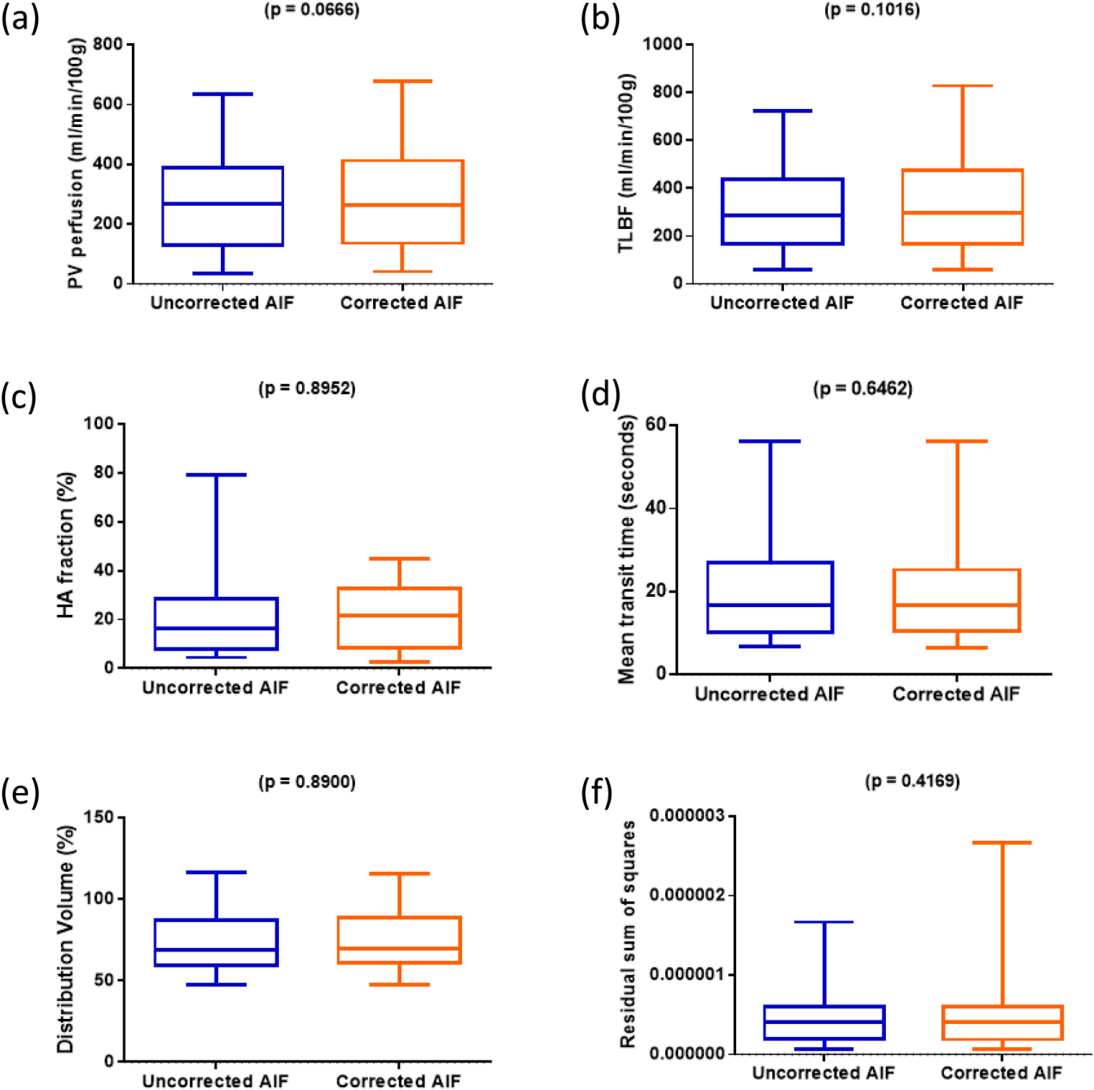
Effects of AIF correction on dual-input single compartment hepatic perfusion parameter estimation. Box and whisker plots for each of the perfusion parameters demonstrate the distribution of (a) PV perfusion, (b) TLBF, (c) HA fraction, (d) MTT, (e) DV and (f) residual sum of squares from pharmacokinetic modelling using uncorrected AIF data on the left and corrected AIF data on the right.

Perfusion parameters and residual sum of squares for model fitting across the sample are presented for uncorrected and corrected AIFs in figure 4 and table 2. DV and residual sum of squares failed normal distribution testing and therefore underwent non-parametric statistical testing. No significant differences were demonstrated between uncorrected and corrected AIF perfusion parameters (PV perfusion mean difference 17.3  ±  8.9 ml min^−1^/100 g, *p*  =  0.0666; TLBF mean difference 19.1  ±  11.1 ml min^−1^/100 g, *p*  =  0.1016; HA fraction mean difference  −0.3  ±  2.0%, *p*  =  0.8952; MTT mean difference  −0.2  ±  1.8 s, *p*  =  0.6462; and DV median difference 0.0%, *p*  =  0.8900) or model fitting (residual sum of squares median difference 2.2  ×  10^−10^, *p*  =  0.4169).

**Table 2.** Perfusion parameters estimated using the dual input single compartment model, with and without cardiac output AIF correction.

	Uncorrected AIF		Corrected AIF		*P*-value
	Mean ± SE	95% CI	Mean ± SE	95% CI	
PV perfusion (ml min^−1^/100 g)	274.3 ± 38.4	(194.3, 354.3)	291.6 ± 41.4	(205.2, 378.0)	0.066
TLBF (ml min^−1^/100 g)	327.5 ± 41.7	(240.6, 414.4)	346.6 ± 46.0	(250.6, 442.7)	0.101
HA fraction (%)	20.7 ± 3.7	(13.0, 28.4)	20.5 ± 2.7	(14.7, 26.2)	0.895
Mean transit time (s)	19.9 ± 2.6	(14.4, 25.4)	19.7 ± 2.7	(14.2, 25.3)	0.646
Distribution volume (%)	73.5 ± 4.0	(65.2, 81.7)	73.7 ± 3.8	(65.7, 81.7)	0.890
Residuals^2^	4.7 × 10^−7^ ± 7.8 × 10^−8^	(2.9 × 10^−7^, 6.2 × 10^−7^)	5.2 × 10^−7^ ± 1.2 × 10^−7^	(2.6 × 10^−7^, 7.8 × 10^−7^)	0.417

*Note*: standard error (SE), confidence interval (CI), with no significant differences demonstrated between uncorrected and corrected AIF perfusion parameters.

### 3.2. Reproducibility studies

**Figure 5.**
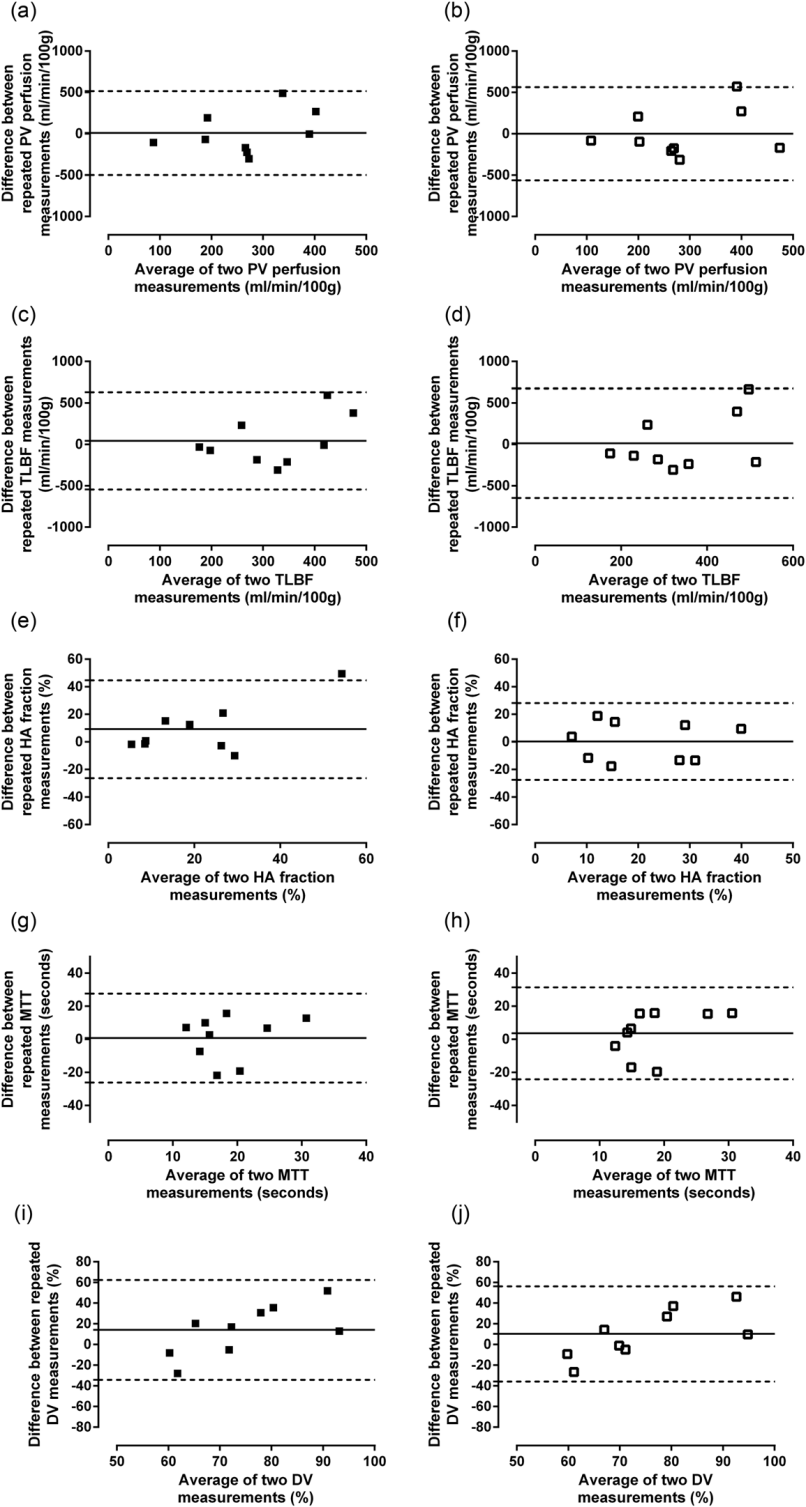
Analysis of agreement of perfusion parameter reproducibility using uncorrected and corrected AIFs with dual-input single compartment modelling. Bland–Altman reproducibility analysis of PV perfusion ((a) and (b)), TLBF ((c) and (d)), HA fraction ((e) and (f)), MTT, ((g) and (h)) and DV ((i) and (j)) using uncorrected ((a), (c), (e), (g) and (i)) and corrected ((b), (d), (f), (h), and (j)) AIFs.

Reproducibility was assessed in 9 normal volunteers 7 d after the initial study (table 3, figure 5). The mean difference for repeated PV perfusion and TLBF measurements was smallest using corrected AIFs (0.92 versus 9.31 ml min^−1^/100 g for corrected and uncorrected AIF PV perfusion and 13.32 versus 43.75 ml min^−1^/100 g for corrected and uncorrected AIF TLBF respectively).

**Table 3.** Summary of reproducibility of perfusion parameters estimated using the dual input single compartment model, with and without cardiac output AIF correction.

	Uncorrected AIF	Corrected AIF
	Dual input single compartment	Dual input single compartment
PV perfusion (ml min^−1^/100 g)		
Mean difference	9.31	**0.92**
BA 95% LoA	** ± 506.1**	±562.8
Coefficient of variation	**64.05%**	65.10%

TLBF (ml min^−1^/100 g)		
Mean difference	43.75	**13.32**
BA 95% LoA	** ± 586.7**	±661.5
Coefficient of variation	**58.29%**	60.85%

HA fraction (%)		
Mean difference	9.26	**0.32**
BA 95% LoA	±35.49	** ± 27.85**
Coefficient of variation	81.71%	**61.36%**

Mean transit time (seconds)		
Mean difference	**2.37**	3.66
BA 95% LoA	** ± 26.89**	±27.79
Coefficient of variation	**60.84%**	61.96%

Distribution volume (%)		
Mean difference	14.14	**10.26**
BA 95% LoA	±48.24	** ± 46.02**
Coefficient of variation	24.66%	**23.92%**

*Note*: emboldened values in the table highlight the best performing method for each statistic.

The BA 95% LoAs for both these perfusion parameters were however smaller using uncorrected AIFs (±562.8 versus  ±506.1 ml min^−1^/100 g for corrected versus uncorrected AIF PV perfusion; and  ±661.5 versus  ±586.7 ml min^−1^/100 g for corrected versus uncorrected AIF TLBF). The CoV for both corrected and uncorrected AIF PV perfusion (65.10% corrected versus 64.05% uncorrected) and TLBF (60.85% corrected versus 58.29% uncorrected) were similar for both methods.

The mean difference between repeated HA fraction measurements was smallest using corrected AIFs (0.32%). This method demonstrated the smallest BA 95% LoAs (±27.85% corrected versus  ±35.49% uncorrected). The CoV was also smallest using corrected AIFs (61.36% corrected versus 81.71% uncorrected).

The smallest mean difference for repeated MTT measurements was demonstrated using uncorrected AIFs (2.37 s) but the smallest mean difference for repeated DV measurements was demonstrated using corrected AIFs (10.26%). Both BA 95% LoAs and coefficients of variation were similar across both methods for both MTT and DV.

## 4. Discussion

The measurement of hepatic vascular parameters has important potential applications in the evaluation of chronic liver disease (Mookerjee 2011) and focal liver lesions (Jackson *et al* 2002). The haemodynamic changes underpinning these conditions however remain poorly understood because of highly invasive methods required for accurate measurement (Chouhan *et al* 2016b).

Liver DCE MRI is a powerful non-invasive tool to investigate pathological haemodynamic changes, but quantification is based on models reliant on measurement of VIFs (Materne *et al* 2002, Annet *et al* 2003, Hagiwara *et al* 2008). Measurement of the AIF using MRI is troublesome and in this study we evaluate a previously proposed method in which independent measurements of cardiac output are used to correct AIFs (Zhang *et al* 2009). Unlike other organs which possess a single afferent blood supply, pharmacokinetic modelling in the liver is uniquely challenging because of reliance on a two separate VIFs. The effects of cardiac output AIF correction are therefore unknown in the liver and this is to our knowledge, the first evaluation of the effects of cardiac output AIF correction on dual-input single compartment modelling for measurement of hepatic perfusion parameters.

We have demonstrated that while the use of cardiac output AIF correction can cause both increases and decreases in sampled AIFs, such corrections on average had no significant effect on estimated hepatic perfusion parameters and model fitting compared to uncorrected data. While AIF correction did reduce the mean difference between perfusion parameters after 7 d (with the exception of MTT), the BA 95% LoA was only improved for HA fraction, with comparable or inferior reproducibility for all other parameters. Furthermore, whilst cardiac output AIF correction improved the HA fraction CoV, the CoV for all other perfusion parameters was comparable to those obtained using uncorrected AIFs.

The attraction of using cardiac output correction is the use of an independently measured patient-specific parameter for correction. Systemic haemodynamic factors such as cardiac output can be altered in chronic liver disease (Mehta *et al* 2014) and while the specific effect of cardiac output changes on hepatic perfusion parameters are unknown, we would argue that individualised correction avoids the potential assumptive errors introduced by using alternative methods such as general population-derived AIFs (Parker *et al* 2006). Cardiac output AIF correction is however reliant on the derivation of a first pass curve using a gamma variate function: an established technique in nuclear medicine, but one that has had limited application to MRI AIFs. The method of AIF correction is also based upon altering the baseline raw SI (${{S}_{0}}$) to a new estimated baseline SI ($S_{0}^{\prime}$), which secondarily affects the AIF CA concentrations throughout the rest of the sampled curve. The method thus addresses errors in AIF sampling that arise from measurement of blood pool T1 and inflow effects, but assumes that the fundamental morphology of the sampled AIF curve is correct. Dephasing (T2^*^) effects at peak AIF CA concentrations—an issue particularly when scanning at higher field strength—are therefore not addressed and remain a major potential source of error (Lee 1991).

Pharmacokinetic modelling is also reliant on sampling of the PVIF as well as the AIF. Correction of one without the other would in principle affect estimated perfusion parameters. Unlike AIF curves, PVIF curves are less prone to VIF sampling errors, demonstrating a slow CA concentration rise, and lower peak CA concentration, compatible with slower, relatively non-pulsatile splanchnic flow. Derivation of a first pass curve using the methods employed would therefore be unfeasible. Correction of the PVIF curve by simply assuming the new estimated baseline SI ($S_{0}^{\prime}$), would only be acceptable if raw baseline SI at the site of AIF and VIF sampling were identical—a phenomenon which is not supported by theory, published data (Dobre *et al* 2007, Zhang *et al* 2013) or the data collected in this study.

While our derived absolute perfusion parameters (PV perfusion and TLBF) are comparable to published DCE MRI data (Aronhime *et al* 2014), these values are still much higher than would be expected physiologically (Soons *et al* 1991, Kuo *et al* 1995, Mehta *et al* 2014). There is also limited published data on the reproducibility of liver DCE MRI using dual-input single compartment modelling (Chouhan *et al* 2016a), but our data demonstrates relatively wide BA 95% LoAs for absolute perfusion parameters. This may reflect natural variation in perfusion, contingent on differences in subject hydration (as supported by the observed reproducibility of data for cardiac output measurements), but may also be secondary to the many other challenges in performing clinical DCE MRI not directly addressed by the present study.

Zhang *et al* were able to use cardiac output AIF correction to demonstrate absolute renal perfusion (glomerular filtration rate) standard deviation reductions and stronger linear correlations across repeated measurements in four subjects (Zhang *et al* 2009). Our findings may reflect the greater variability introduced by dual-input modelling, and also highlight that PVIFs (the main contributor to PV and therefore TLBF perfusion) were unchanged for modelling of both uncorrected and cardiac output corrected data. Finally, it is worth noting that the overall study size was small. Arterial contributions to hepatic perfusion are small in healthy tissue, thereby further compromising study power.

This study adds to current knowledge by evaluating a previously proposed method for correcting a recognised source of error in DCE MRI pharmacokinetic modelling, in the context of liver perfusion measurement. Errors in MRI AIF sampling remain an important limitation of DCE MRI in the liver and elsewhere in the body. Evaluation of the benefits or otherwise of cardiac output correction on liver DCE MRI perfusion parameter estimation is important if we are to determine robust strategies for accurate and reproducible liver DCE MRI in the clinical setting. Our data suggests that the clinical value of cardiac output AIF correction for DCE of the liver is debatable: the time taken for acquisition and analysis of PCMRI cardiac output measurements, combined with the added complexity involved in correcting the AIF are important barriers to implementation. It could be argued that the improved reproducibility and reduced CoV of HA fraction is an important advantage, outweighing the apparent deleterious effects on the reproducibility of estimated absolute perfusion parameters (PV perfusion and TLBF), particularly in the context of general overestimation of PV perfusion and TLBF by DCE MRI. Increases in relative arterialisation of tissues (as measured by HA fraction) are important pathophysiological sequelae of chronic liver disease and focal malignant lesions, thus underlining the potential value of using cardiac output AIF correction for measurement of HA fraction.

## 5. Conclusion

AIFs correction using PCMRI aortic flow measurements has limited effect on estimated dual-input single compartment hepatic perfusion parameters and does not improve the reproducibility of PV perfusion and TLBF measurements. AIF correction does however have apparent advantages in improving the reproducibility of HA fraction. This finding has potential importance because arterialisation of liver perfusion is increased in chronic liver disease and within malignant liver lesions.
